# Emergence of microbial infections in some hospitals of Cairo, Egypt: studying their corresponding antimicrobial resistance profiles

**DOI:** 10.1186/s12879-023-08397-4

**Published:** 2023-06-22

**Authors:** Asmaa K. Helmy, Nagwa M. Sidkey, Ramy E. El-Badawy, Ahmed G. Hegazi

**Affiliations:** 1 Botany and Microbiology Department, Faculty of Science for Girls, Al-Azhar University, Cairo, Egypt; 2 Medical lab specialist, Cleopatra hospital, Cairo, Egypt; 3 Zoonotic Diseases Department, National Research Centre, Dokki, Giza Egypt

**Keywords:** Microbial infections, COVID-19, MDR, Glycylcycline, Nystatin

## Abstract

**Background:**

Antimicrobial resistance is one of the ten major public health threats facing humanity, especially in developing countries. Identification of the pathogens responsible for different microbial infections and antimicrobial resistance patterns are important to help clinicians to choose the correct empirical drugs and provide optimal patient care.

**Methods:**

During the period from November 2020 to January 2021, one hundred microbial isolates were collected randomly from different specimens from some hospitals in Cairo, Egypt. Sputum and chest specimens were from COVID-19 patients. Antimicrobial susceptibility testing was performed according to CLSI guidelines.

**Results:**

Most microbial infections were more common in males and in elderly people over 45 years of age. They were caused by Gram-negative, Gram-positive bacteria, and yeast isolates that represented 69%, 15%, and 16%, respectively. Uropathogenic *Escherichia coli* (35%) were the most prevalent microbial isolates and showed high resistance rates towards penicillin, ampicillin, and cefixime, followed by *Klebsiella* spp. (13%) and *Candida* spp. (16%). Of all microbial isolates, *Acinetobacter* spp., *Serratia* spp., *Hafnia alvei*, and *Klebsiella ozaenae* were extremely multidrug-resistant (MDR) and have resisted all antibiotic classes used, except for glycylcycline, in varying degrees. *Acinetobacter* spp., *Serratia* spp., and *Candida* spp. were secondary microbial infections in COVID-19 patients, while *H. alvei* was a bloodstream infection isolate and *K. ozaenae* was recorded in most infections. Moreover, about half of *Staphylococcus aureus* strains were MRSA isolates and reported low rates of resistance to glycylcycline and linezolid. In comparison, *Candida* spp. showed high resistance rates between 77 and 100% to azole drugs and terbinafine, while no resistance rate towards nystatin was reported. Indeed, glycylcycline, linezolid, and nystatin were considered the drugs of choice for the treatment of MDR infections.

**Conclusion:**

The prevalence of antimicrobial resistance in some Egyptian hospitals was high among Gram-negative, Gram-positive bacteria, and *candida* spp. The high resistance pattern —especially in secondary microbial infections in COVID-19 patients— to most antibiotics used is a matter of great concern, portends an inevitable catastrophe, and requires continuous monitoring to avoid the evolution of new generations.

## Introduction

Nosocomial or healthcare-associated infections caused by antimicrobial-resistant pathogens represent a serious burden and ongoing threat to patients’ health and safety [1]. The prevalence of nosocomial infection varies from one setting to another depending on the level of development of the health system, since it is more prevalent in developing countries compared to developed ones and is associated with different risk factors [2]. During the coronavirus disease 2019 (COVID-19) pandemic, changes in hospital infection prevention and control and antibiotic stewardship strategies have had implications for nosocomial infection rates and antimicrobial resistance [3].

Antimicrobial resistance is a growing problem that causes over 700,000 deaths every year around the world [4] and is expected to cause the deaths of 10 million people by 2050 [5]. The excessive use of antibiotics or antifungals, empirical treatment without antimicrobial susceptibility testing and self-treatment lead to mutation and increased drug resistance [6]. Reporting of susceptibility testing results is a key reference to choose the correct antimicrobial and avoiding the emergence of new antimicrobial resistance. In Egypt, the most common nosocomial infections are urinary tract, wound, respiratory tract, and bloodstream infections [7]. Nosocomial infections were caused by microbes, which includ bacteria, viruses, and fungi [7, 8]. The most common bacterial pathogens included *E. coli, P. aeruginosa, Klebsiella* spp., *Enterobacter* spp., *Proteus* spp., *Serratia* spp., *Acinetobacter* spp., *S. aureus*, Coagulase Negative Staphylococci (CoNS), and *Streptococcus* spp. [9]. *Acinetobacter baumanii* is linked to a high mortality rate in the intensive care units because of its inherent MDR properties [8]. Fungal pathogens are most commonly found in immune-compromised patients and those who have indwelling devices, such as urinary catheters and central lines. *Candida* species, such as *C. albicans, C. glabrata*, and *C. parapsilosis* as well as *Aspergillus* species, are the most prevalent causes of fungal infection [8, 10]. Therefore, the present study is a trail to give a broad picture of pathogens responsible for different infections and the antimicrobial resistance of many bacterial and yeast isolates.

## Methods

### Study design

The data of microbial isolates from different clinical specimens were collected from the Clinical Microbiology Department at some hospitals in Cairo, Egypt, during the period from November 2020 to January 2021. Patient samples in this study were included and analyzed by sex and age.

### Isolation and identification of the microbial isolates

Microbial isolates were collected randomly and in aseptic conditions from different clinical specimens, including urine, blood, wound swab, abscess swab, liver pus, ascites swab, pelvic aspiration, pleural effusion, vaginal swab, as well as sputum swab and chest aspirations from COVID-19 patients.

The clinical specimens were cultured immediately after collection on Blood and MacConkey agar, except urine specimens, which were cultured on Blood and CLED agar medium according to the standard method and incubated for 18–24 h under standard conditions at 37 °C [11]. After the incubation period, the different colonies of bacteria on Blood, CLED, and MacConkey agar were sub-cultured on nutrient agar medium in order to purify the isolated pathogens, while yeast-like isolates were sub-cultured on CHROM agar medium [12]. All media used in the present study were from Oxoid, UK. Pure isolates of bacteria and yeast were subjected to the Gram-staining technique, examined microscopically, and finally identified by VITEK 2 system [13].

### Antimicrobial susceptibility testing

Antibiotic sensitivity testing of bacterial isolates was performed for commonly used antibiotics by the standard disc diffusion technique on Muller-Hinton agar according to the Kirby-Bauer method [14]. After the incubation period at 37°C, the zone of inhibition was measured, and results were interpreted according to the Clinical and Laboratory Standards Institute [15]. Due to the lack of established CLSI breakpoints for tigecycline at this time FDA breakpoints were: (susceptible at MIC ≤ 2 mg/l, with zone diameter ≥ 19 mm; intermediate at MIC ≥ 4 mg/l, with zone diameter resistant 15–18 mm; resistant at MIC ≥ 8 mg/l, with zone diameter ≥ 14 mm.” https://www.accessdata.fda.gov/drugsatfda_docs/label/2013/021821s026s031lbl.pdf.

Twenty-four antibiotics were used against Gram-negative bacterial isolates and twenty-eight antibiotics were tested against Gram-positive bacterial isolates. The antibiotics used in this respect were the following classes: (I) Penicillins: penicillin (P 10), ampicillin (AMP 10), amoxicillin-clavulanic acid (AMC 30) and sulbactam/ampicillin (SAM 20), (II) Cephalosporins: ceftazidime (CAZ 30), cefixime (CFM 5), cefoperazone (CFP 75), ceftriaxone (CRO 30), cefotaxime (CTX 30) and cefepime (FEP 30), (III) DNA synthesis inhibitors (fluoroquinolones): ciprofloxacin (CIP 5), norfloxacin (NOR 10) and ofloxacin (OFX 5), (IV) Protein Synthesis Inhibitors: amikacin (AK 30), gentamicin (CN 10), tigecycline (TGC 15) and chloramphenicol (CL 30), (V) Carbapenems: ertapenem (ETP 10) and meropenem (MEM 10), (VI) Others: ceftazidime-avibactam (CZA 50), ceftolozane/tazobactam (C/T), cefoperazone/sulbactam (SCF 105), sulphamethoxazole/trimethoprim (SXT 25), piperacillin/tazobactam (TZP 110), (VII) Gram-positive antibiotics: clindamycin (DA 2), erythromycin (E 15), linezolid (LZD 30), and oxacillin (OX 1).

Various commonly used antifungals were tested on yeast isolates on 2% glucose-supplemented Mueller-Hinton agar [16]. The antifungal discs used in this test were the following: nystatin (100 U), clotrimazole (10 µg), fluconazole (25 µg), itraconazole (10 µg) and terbinafine (1 µg). The results were explained using the standard zone sizes of the Clinical and Laboratory Standards Institute guidelines [17].

### Data analysis

Data were entered and analyzed using Statistical Package for Social Science version 27 (IBM Corp released 2020.IBM SPSS statistics. Armonk, NY: IBM Corp). A Chi-square test was used for the comparison between groups, and a *P value* lower than 0.05 was regarded as statistically significant.

## Results

### Percentage of microbial isolates in relation to the source of microbial infection

**Fig. 1:**
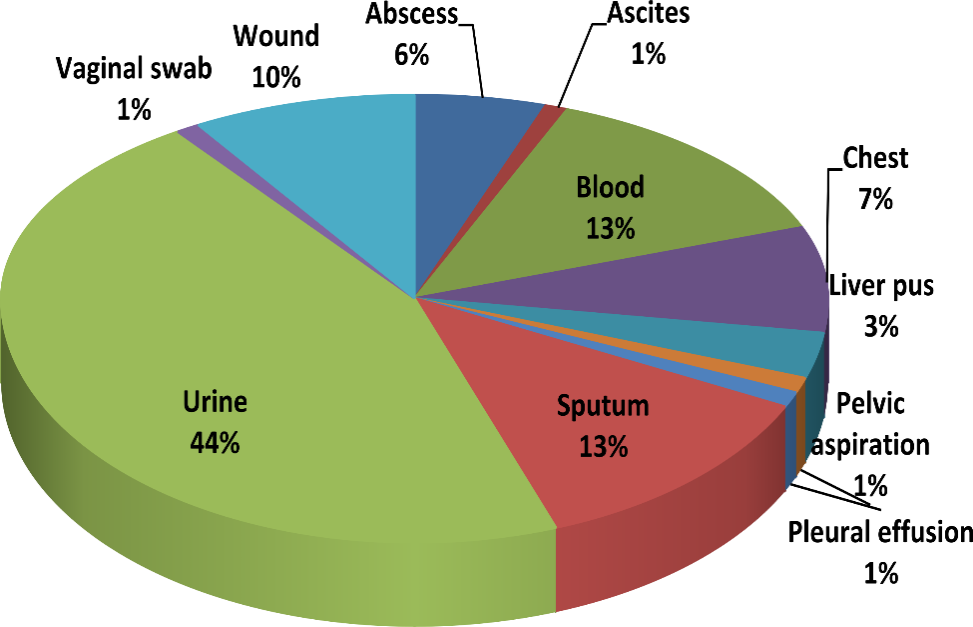
Percentage of the microbial isolates in relation to the isolation source

One hundred (100) clinical pathogens were isolated from different patients from different specimens, as illustrated in Fig. 1. The most common source of microbial infection was in urine specimens with a percentage of 44%, followed by 20% COVID-19 patients’ isolates (13% sputum and 7% chest isolates), 13% blood, and 10% wound isolates.

### Prevalence of microbial Infections in relation to patients’ gender

**Fig. 2:**
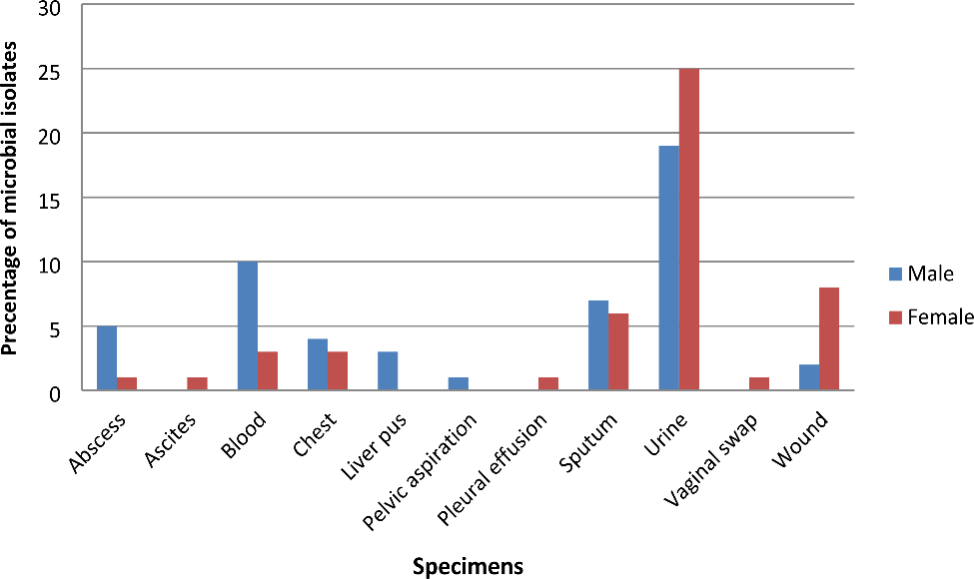
The prevalence of microbial infections among patients’ gender

From the results displayed in Table 1; Fig. 2, the distribution of microbial infections among patients increased in males (51%) than females (49%) (*P value* = 0.054 > 0.05), although the differences were not statistically significant. Urinary infections were more common in females than males, however none statistically significant difference was found (*P* = 0.166 > 0.050) as well as abscess, chest, and sputum infection showed none statistically significant difference. On the other hand, blood infection was more prevalent in males (*P value =* 0.045 < 0.050), and wound infection was more prevalent in females (*P value* = 0.039 < 0.050), so both results were statistically significant.

**Table 1:** The prevalence of microbial infections among patients’ gender

Specimens	Number (%)	patients’ gender		*p- value*
		Male n (%)	Female n (%)	
Abscess	6 (6)	5 (83.3)	1 (16.7)	0.102
Ascites	1 (1)	0	1 (100)	NA
Blood	13 (13)	10 (76.9)	3 (23.1)	0.045
Chest	7 (7)	4 (57.1)	3 (42.9)	0.736
Liver pus	3 (3)	3 (100)	0	NA
Pelvic aspiration	1 (1)	1 (100)	0	NA
Pleural effusion	1 (1)	0	1 (100)	NA
Sputum	13 (13)	7 (53.8)	6 (46.2)	0.825
Urine	44 (44)	19 (43.2)	25 (56.8)	0.166
Vaginal swab	1 (1)	0	1 (100)	NA
Wound	10 (10)	2 (20)	8 (80)	0.039
**Total (%)**	**100 (100)**	**51 (51)**	**49 (49)**	0.054

NA = not applicable

### Prevalence of microbial Infections among patients’ age groups

**Fig. 3:**
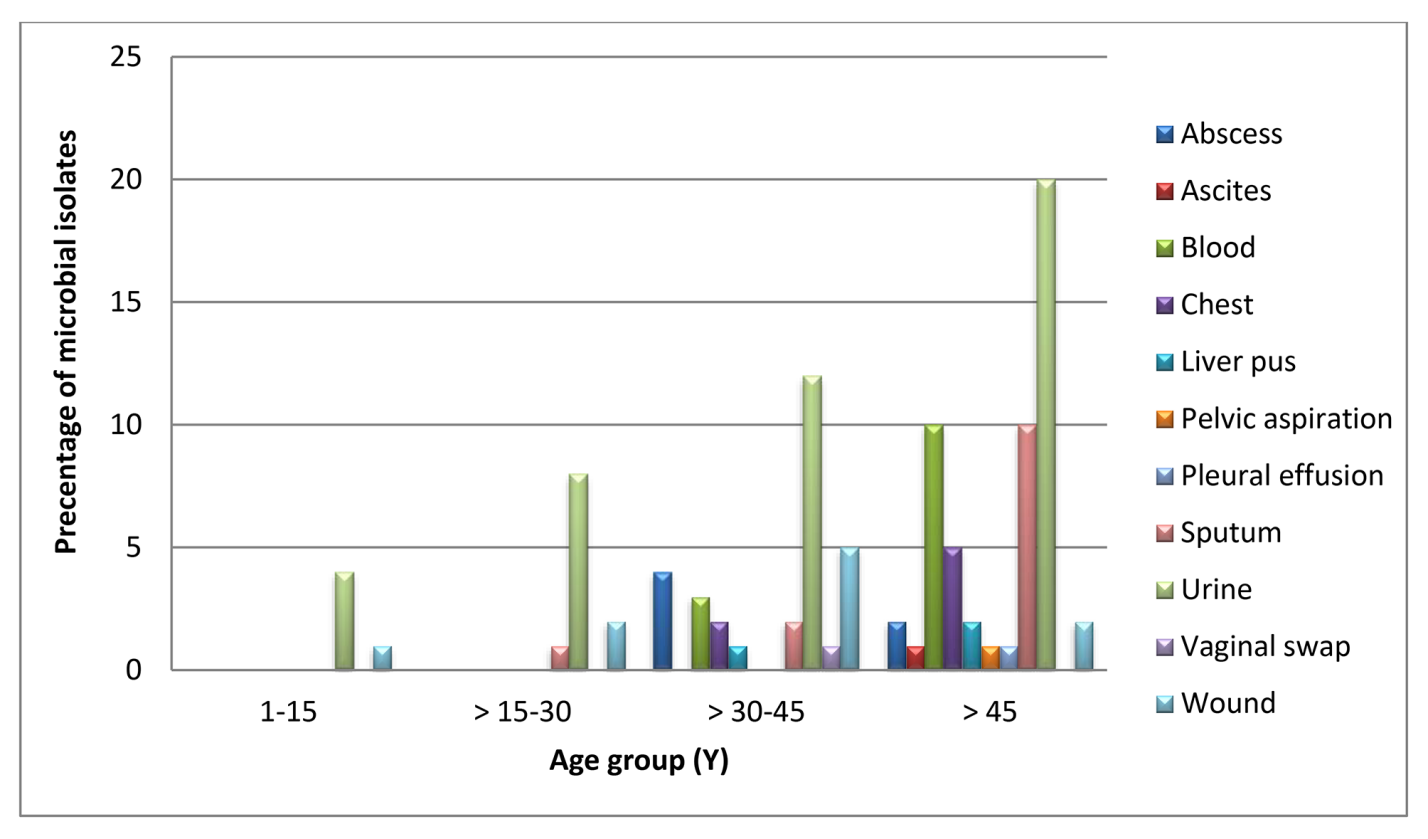
Distribution of microbial infections in relation to patients’ age groups

Patients in this study were separated into 4 age groups from infants to old patients (1 − 93 years). Most microbial infections were found in the age group over 45 years and represented about 54% of microbial infections (P = 0.055), so P > 0.05 and a non-significant difference was found as shown in Table 2; Fig. 3.

**Table 2:** Distribution of microbial infections in relation to patients’ age groups

Specimens	Number (%)	Age group (Y)				*p- value*
		1–15	> 15–30	> 30–45	> 45	
Abscess	6 (6)	0	0	4 (66.7)	2 (33.3)	0.223
Ascites	1 (1)	0	0	0	1 (100)	NA
Blood	13 (13)	0	0	3 (30.1)	10 (76.9)	NA
Chest	7 (7)	0	0	2 (28.6)	5 (71.4)	0.652
Liver pus	3 (7)	0	0	1 (33.3)	2 (66.7)	NA
Pelvic aspiration	1 (1)	0	0	0	1 (100)	NA
Pleural effusion	1 (1)	0	0	0	1 (100)	NA
Sputum	13 (13)	0	1 (9.09)	2 (6.7)	10 (76.9)	0.332
Urine	44 (44)	4 (4.1)	8 (18.1)	12 (27.3)	20 (45.5)	0.055
Vaginal swabs	1 (1)	0	0	1 (100)	0	NA
Wound	10 (10)	1 (10)	2 (20)	5 (50)	2 (20)	0.155
**Total**	**100 (100)**	**5 (5)**	**11 (11)**	**30 (30)**	**54 (45)**	0.645

NA = not applicable

### Characterization and identification of the microbial isolates

**Fig. 4:**
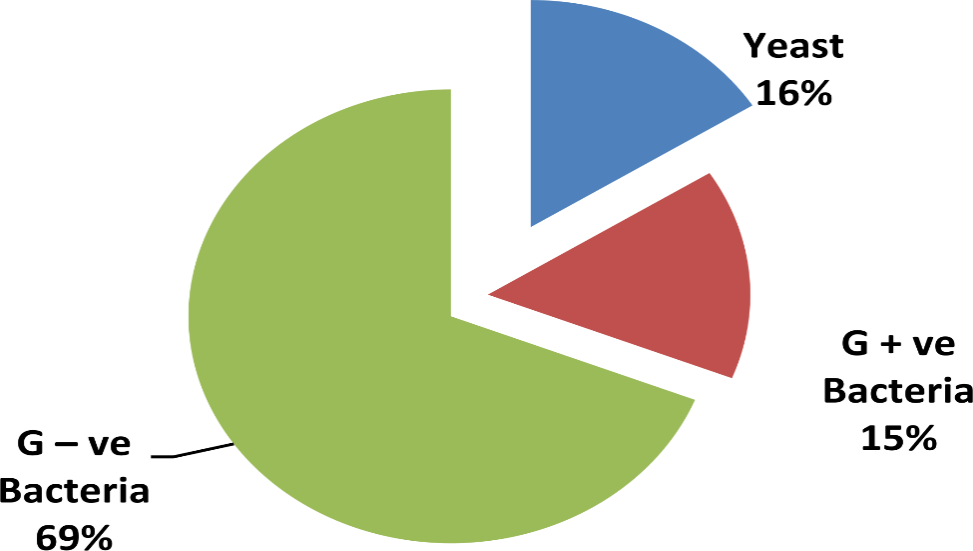
Prevalence of microbial isolates according to gram staining and domain

The microbial isolates were subjected to microscopic examination, with cell shape and arrangement being bacilli, cocci, coccobacilli, coccoid cluster, and ovoid in shape. Additionally, the Gram-staining technique was performed and reported 15% Gram-positive bacterial isolates, 69% Gram-negative bacterial isolates and 16% Gram-positive staining yeast isolates that appeared in an oval shape under the microscope, as shown in Fig. 4.

**Fig. 5:**
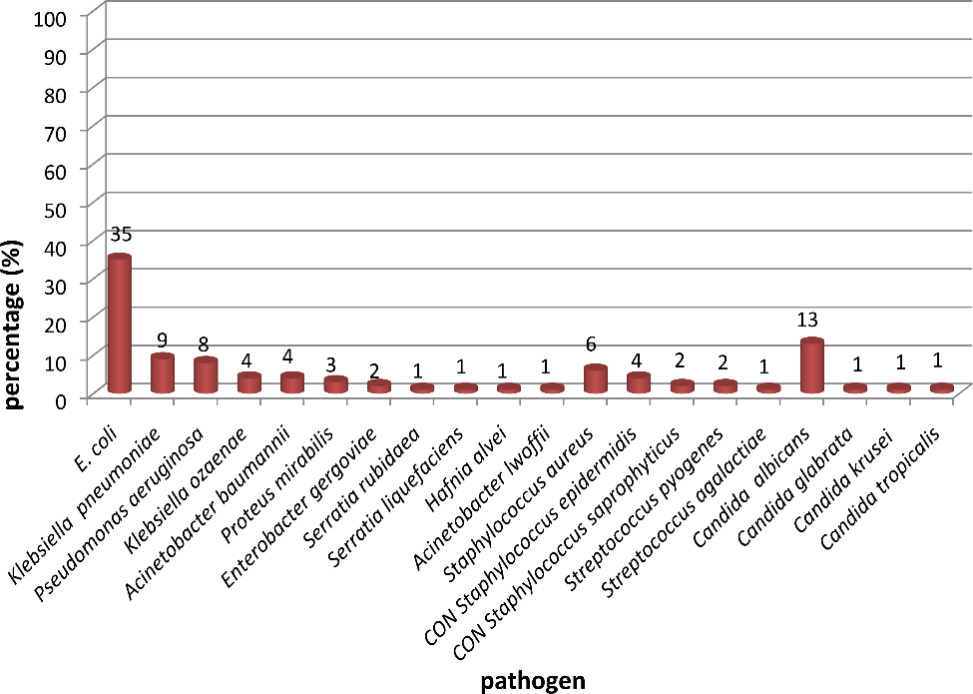
Percentage of the identified microbial isolates

The microbial isolates were identified according to phenotypic and biochemical characteristics by VITEK 2 system. In view of the presented data from Fig. 5, it was observed that *E. coli* was the most frequently identified Gram-negative bacteria (35%), followed by *Klebsiella* spp. (13%) (*Klebsiella pneumoniae* 9% and *Klebsiella ozaenae* 4%), *Pseudomonas aeruginosa* (8%), *Acinetobacter* spp. (5%) (*Acinetobacter baumannii* 4% and *Acinetobacter lwoffii* 1%), *Proteus mirabilis* (3%), *Enterobacter gergoviae* (2%), *Serratia* spp. (2%) (*Serratia rubidaea* 1% and *Serratia liquefaciens* 1%), and *Hafnia alvei* (1%). Moreover, among the Gram-positive bacterial isolates, *Staphylococcus aureus* and CON *Staphylococcus* spp. were most frequently identified, representing 6% for each, followed by *Streptococcus pyogenes* (2%) and *Streptococcus agalactiae* (1%). On the other hand, the non-bacterial growth isolates were *Candida* spp., including *Candida albicans, Candida glabrata, Candida krusei*, and *Candida tropicalis*, which represented 13, 1, 1, and 1%, respectively, of all microbial isolates.

### Distribution of the identified microbial isolates among different specimens

The data in Table 3 indicated that urine specimens were the most common source of microbial infections, accounting for 44% of microbial infections. Uropathogenic *E. coli* 27% was the most frequently identified Gram-negative bacteria in urine specimens, followed by *Klebsiella* spp. (5%), *P. aeruginosa* (2%), and *P. mirabilis* (2%) as well as CON *S. agalactiae* (1%), the only identified Gram-positive bacteria. Additionally, *C. albicans* (6%) and *C. krusei* (1%) were the identified yeast isolates.

On the other hand, sputum and chest swabs microbial isolates were secondary infection in COVID-19 patients and accounted for 20% of microbial infections. Gram-negative bacteria (*Acinetobacter* spp., *P. aeruginosa*, *Klebsiella* spp., and *Serratia* spp.) were the most prevalent sputum and chest isolates, followed by *Candida* spp., while Gram-positive bacteria represented by *S. aureus* were the least prevalent.

Blood infections accounted for 13% of microbial isolates and were caused by both Gram-positive and Gram-negative bacteria as well as by certain yeast (*C. albicans*). The most commonly encountered bacteria were Gram-positive, represented by *S. aureus* and CON *Staphylococcus* spp., followed by Gram-negative bacteria that included *E. gergoviae*, *E. coli*, *H. alvei*, and *K. ozaenae.*

Moreover, wound infections from wound swab specimens represented 10% of microbial infections and were caused by both Gram-positive (*S. aureus*, CONS *epidermidis*, and *S*. *pyogenes*) and Gram-negative bacteria (*E. coli* and *P. aeruginosa*), while yeast were not identified in our study.

**Table 3:** Distribution of the identified microbial isolates on basis of the source of microbial isolates

pathogen	%	Urine	Sputum	Chest	Blood	Wound	Abscess	Others
Gram-negative bacteria								
*A. baumannii*	4	-	1	2	-	-	-	1
* A. lwoffii*	1	-	-	1	-	-	-	-
*E. coli*	35	27	-	-	1	2	3	2
*E. gergoviae*	2	-	-	-	2	-	-	-
*H. alvei*	1	-	-	-	1	-	-	-
*K. pneumoniae*	9	4	2	-	-	-	-	3
* K. ozaenae*	4	1	1	-	1	-	1	-
*P. mirabilis*	3	2	-	-	-	-	1	-
*P. aeruginosa*	8	2	2	2	-	2	-	-
*S. liquefaciens*	1	-	1	-	-	-	-	-
*S. rubidaea*	1	-	-	1	-	-	-	-
**Gram-positive bacteria**								
*S. aureus*	6	-	1	-	3	2	-	-
CON *S. epidermidis*	4	-	-	-	2	2	-	-
CON *S. saprophyticus*	2	-	-	-	1	-	1	-
*S. pyogenes*	2	-	-	-	-	2	-	-
*S. agalactiae*	1	1	-	-	-	-	-	-
**Yeast**								
*C. albicans*	13	6	4	-	2	-	-	1
* C. glabrata*	1	-	1	-	-	-	-	-
*C. krusei*	1	1	-	-	-	-	-	-
*C. tropicalis*	1	-	-	1	-	-	-	-
**Total**	**100**	**44**	**13**	7	**13**	**10**	**6**	**7**

### Antimicrobial resistance pattern

#### Resistance pattern in Gram-negative bacterial isolates

Twenty-four antibiotics were tested against Gram-negative bacterial isolates. Data in Table 4 revealed the resistance percentage of different Gram-negative bacterial isolates to different antibiotics used.

Different microbial infection isolates of *Acinetobacter* spp., *S. rubidaea*, and *S. liquefaciens* were secondary microbial infection from COVID-19 patients and showed high resistance rates between 80% and 100% towards different classes of antibiotics, including penicillins, cephalosporins, fluoroquinolones, protein synthesis inhibitors (amikacin, gentamicin, and chloramphenicol), carbapenems, and others combined antibiotics (ceftolozane/tazobactam, sulphamethoxazole/trimethoprim, and piperacillin/tazobactam). Additionally, they showed complete sensitivity towards glycylcycline.

*H. alvei* (blood isolate) and *K. ozaenae* from different specimens (urine, sputum, blood, and abscess isolates) have also complete resistance rates of 100% to most classes of antibiotics used, except for glycylcycline in *K. ozaenae* that was completely sensitive.

The majority of *K. pneumoniae* were urine and sputum clinical isolates. They reported complete resistance of 100% to penicillin, ampicillin, cefixime, and sulphamethoxazole/trimethoprim antibiotics as well as complete sensitivity of 100% to glycylcycline. In addition, resistance rates over 65% have been reported in sulbactam/ampicillin, cephalosporins (cefoperazone, ceftriaxone, cefotaxime, and cefepime), fluoroquinolones (ciprofloxacin, norfloxacin, and ofloxacin), and chloramphenicol. In comparison, low rates of resistance to carbapenems and combined antibiotics have been reported.

Indeed, *E. coli* showed resistance to penicillin, ampicillin, cefixime, and chloramphenicol at different rates of 100, 97, 89, and 46%, respectively. Furthermore, about third of isolates were resistant to amoxicillin-clavulanic acid, sulphamethoxazole/trimethoprim, cephalosporins (except for cefixime), and fluoroquinolones. In contrast, low resistance rates were reported towards carbapenems group, chloramphenicol, glycylcycline, and combined antibiotics.

Among the isolates, *E. gergoviae* were blood isolates and showed sensitivity to most of the used antibiotics, with the exception of penicillin, ampicillin, cefixime, and chloramphenicol. Moreover, half of the isolates were resistant to gentamicin and sulphamethoxazole/trimethoprim.

Additionally, *P. mirabilis* isolates were found in urine and abscesses specimens. They showed resistance rates between 67% and 100% to penicillin, ampicillin, sulphamethoxazole/trimethoprim, norfloxacin, ofloxacin, and gentamicin, while they had a resistance rate of 33% towards amoxicillin-clavulanic acid, sulbactam/ampicillin, cefixime, ciprofloxacin, chloramphenicol, ertapenem, and meropenem. On the other hand, *P. mirabilis* isolates were completely sensitive to cephalosporins.

The microbial isolates of *P. aeruginosa* were found in most specimens that included urine, blood, chest, and sputum isolates. All isolates were resistant to penicillins group, cefixime, chloramphenicol, and sulphamethoxazole/trimethoprim, while showing resistance rates between 50% and 75% towards cefoperazone, ceftriaxone, cefotaxime, cefepime, norfloxacin, ofloxacin, glycylcycline, ertapenem, and meropenem. In comparison, *P. aeruginosa* isolates showed a low resistance rate of 25% towards ceftazidime, ceftazidime-avibactam, and ceftolozane/tazobactam.

**Table 4:** Resistance degree of Gram-negative bacterial isolates to each antibiotic used

Antibiotic	*Acinetobacter* spp.	*E. coli*	*E. gergoviae*	*H. alvei*	*K. pneumoniae*	*K. ozaenae*	*P.* *Mirabilis*	*P. aeruginosa*	*Serratia* spp.
Penicillins group									
Penicillin **(P 10)**	**100**	**100**	**100**	**100**	**100**	**100**	**100**	**100**	**100**
Ampicillin **(AMP 10)**	**100**	**97**	**100**	**100**	**100**	**100**	**100**	**100**	**100**
Amoxicillin-clavulanic acid **(AMC 30)**	**100**	34	50	**100**	**56**	**100**	33	**100**	**100**
Sulbactam/ampicillin **(SAM 20)**	**100**	31	0	**100**	**67**	**100**	33	**100**	**100**
**Cephalosporins group**									
Ceftazidime **(CAZ 30)**	**100**	14	0	**100**	**56**	**100**	0	25	**100**
Cefixime **(CFM 5)**	**100**	**89**	**100**	**100**	**100**	**100**	33	**100**	**100**
Cefoperazone **(CFP 75)**	**80**	31	0	**100**	**78**	**100**	0	**75**	**100**
Ceftriaxone **(CRO 30)**	**100**	20	0	**100**	**78**	**100**	0	**63**	**100**
Cefotaxime **(CTX 30)**	**100**	23	0	**100**	**78**	**100**	0	**75**	**100**
Cefepime **(FEP 30)**	**100**	26	0	**100**	**78**	**100**	0	**63**	**100**
**DNA synthesis Inhibitors (Fluoroquinolones group)**									
Ciprofloxacin **(CIP 5)**	**80**	26	0	**100**	**67**	**100**	33	38	**100**
Norfloxacin **(NOR 10)**	**100**	26	0	**100**	**78**	**100**	**67**	**63**	**100**
Ofloxacin **(OFX 5)**	**100**	26	0	**100**	**78**	**100**	**67**	**75**	**100**
**Protein Synthesis Inhibitors**									
Amikacin **(AK 30)**	**100**	6	0	**100**	44	**100**	0	25	**100**
Gentamicin **(CN 10)**	**80**	17	50	**100**	44	**100**	**67**	38	**100**
Glycylcycline **(TGC 150)**	**0**	0	0	**100**	0	0	0	**63**	0
Chloramphenicol **(CL 30)**	**100**	**46**	**100**	**100**	**89**	**100**	33	**100**	**100**
**Carbapenems group**									
Ertapenem **(ETP 10)**	**100**	3	0	**100**	33	**100**	33	**63**	**100**
Meropenem **(MEM 10)**	**100**	3	0	**100**	33	**100**	33	**50**	**100**
**Others**									
Ceftazidime-avibactam **(CZA 50)**	**100**	0	0	**100**	22	20	0	25	**50**
Ceftolozane/tazobactam **(C/T)**	**80**	6	0	**100**	22	**100**	0	25	**100**
Cefoperazone/Sulbactam **(SCF 105)**	**80**	3	0	**100**	33	**100**	0	38	**100**
Sulphamethoxazole/trimethoprim **(SXT 25)**	**100**	37	50	**100**	**100**	**100**	**100**	**100**	**100**
Piperacillin/tazobactam **(TZP 110)**	**80**	3	0	**100**	22	**100**	0	38	**100**

#### Resistance pattern in Gram-positive bacterial isolates

Twenty-eight antibiotics were tested against Gram-positive bacterial isolates. Data in Table 5 revealed the resistance percentage of different Gram-positive bacterial isolates to different antibiotics used.

Most of *S. aureus* strains were blood and wound microbial isolates, with about half of the isolates being methicillin-resistant *S. aureus* (MRSA) strains. The microbial isolates of *S. aureus* showed a high resistance rate of 83.0% towards penicillin, ampicillin, and cefixime, while they showed moderate resistance (50%) to oxacillin, amoxicillin-clavulanic acid, ceftazidime, cefotaxime, fluoroquinolones (ciprofloxacin, norfloxacin, and ofloxacin), chloramphenicol, carbapenems (ertapenem and meropenem), ceftolozane/tazobactam, and sulphamethoxazole/trimethoprim.

The CON *Staphylococcus* spp. detected in our study were blood, wound, and abscess infection isolates, namely, *S. epidermidis* and *S. saprophyticus*. They showed high resistance rates of 100% to penicillin, 83% to ampicillin and cefixime both, and 50% to clindamycin, erythromycin, and oxacillin, while low rates of resistance were reported towards amoxicillin-clavulanic acid, sulbactam/ampicillin, cephalosporins (ceftazidime, cefoperazone, ceftriaxone, cefotaxime, and cefepime), fluoroquinolones, carbapenems, sulphamethoxazole/trimethoprim, and linezolid.

*Streptococcus* spp. microbial isolates in the present study included *S. agalactiae* (group B streptococcus) urine isolates and *S. pyogenes* (group A streptococcus) wound infection isolates. They showed complete sensitivity to most classes of antibiotics used, with the exception of penicillin, ampicillin, cefixime, amikacin, gentamicin, and sulphamethoxazole/trimethoprim, which recorded low resistant rates of 33%.

**Table 5:** Resistance degree of Gram-positive bacterial isolates to each antibiotic used

Antibiotic	*S. aurus*	CON *Staphylococcus*	*Streptococcus* spp.
Penicillins group			
Penicillin **(P 10)**	**83**	**100**	33
Ampicillin **(AMP 10)**	**83**	**83**	33
Amoxicillin-clavulanic acid **(AMC 30)**	**50**	33	0
Sulbactam/ampicillin **(SAM 20)**	33	17	0
**Cephalosporins group**			
Ceftazidime **(CAZ 30)**	**50**	17	0
cefixime **(CFM 5)**	**83**	**83**	33
Cefoperazone **(CFP 75)**	33	17	0
Ceftriaxone **(CRO 30)**	33	33	0
Cefotaxime **(CTX 30)**	**50**	33	0
Cefepime **(FEP 30)**	33	33	0
**DNA synthesis Inhibitors (Fluoroquinolones)**			
Ciprofloxacin **(CIP 5)**	**50**	33	0
Norfloxacin **(NOR 10)**	**50**	33	0
Ofloxacin **(OFX 5)**	**50**	33	0
**Protein Synthesis Inhibitors**			
Amikacin **(AK 30)**	0	0	33
Gentamicin **(CN 10)**	17	17	33
Glycylcycline **(TGC 150)**	0	0	0
Chloramphenicol **(CL 30)**	**50**	33	0
**Carbapenems group**			
Ertapenem **(ETP 10)**	**50**	33	0
Meropenem **(MEM 10)**	**50**	17	0
**Others**			
Ceftazidime-avibactam **(CZA 50)**	33	17	0
Ceftolozane/tazobactam **(C/T)**	**50**	17	0
Cefoperazone/Sulbactam **(SCF 105)**	17	0	0
Sulphamethoxazole/trimethoprim **(SXT 25)**	**50**	33	33
piperacillin/tazobactam **(TZP 110)**	17	17	0
**Gram-positive antibiotics**			
Clindamycin **(DA 2)**	33	**50**	0
Erythromycin **(E 15)**	33	**50**	0
Linezolid **(LZD 30)**	17	17	0
Oxacillin **(OX 1)**	**50**	**50**	0

#### Resistance pattern in *Candida* spp. microbial isolates

Five antifungals were tested against *Candida* spp. isolates on 2% glucose enriched Mueller-Hinton agar. *Candida* spp. showed a high resistance rate to azole drugs (fluconazole 88%, itraconazole 81%, and clotrimazole 75%) and terbinafine 81%. On the other hand, they were completely sensitive to polyene antifungal medication (nystatin) as indicated in Table 6.

**Table 6:** Resistance degree of *Candida* spp. isolates to different antifungals used

*Candida* spp.	Antifungal resistance rate (%)				
	Nystatin 100 U	Clotrimazole 10 µg	Fluconazole 25 µg	Itraconazole 10 µg	Terbinafine 1 µg
***C. albicans*** **(n = 16)**	0	77	85	85	85
*** C. glabrata*** **(n = 1)**	0	100	100	100	100
*** C. krusei*** **(n = 1)**	0	100	100	100	100
*** C. tropicalis*** **(n = 1)**	0	0	100	0	0
**Total (n = 16)**	**0**	**75**	**88**	**81**	**81**

## Discussion

Healthcare-associated infection is a major problem in healthcare facilities and is associated with increased morbidity, mortality, prolonged hospital stays, and increased antimicrobial resistance [7, 18]. Over recent years, extensive exposure to antimicrobials has led to the emergence and widespread of MDR pathogens with developed mechanisms of resistance against β-lactams, cotrimoxazole, sulfamethoxazole/trimethoprim, nitrofurantoin, carbapenems, and fluoroquinolones [2, 4, 6].

In the current study, urine specimens were the most prevalent source of microbial infections, followed by COVID-19 patients**’** specimens from sputum and chest swabs, blood, and wound infections. Urinary tract infection (UTI) is among the most common community- and hospital-associated microbial infections, affecting about 150 million people worldwide each year [19]. Chest and bloodstream infections are common conditions causing death and morbidity in humans of all ages, with a high burden on public health. These infections are frequent and present life-threatening conditions in hospital settings [8, 20]. Additionally, other studies reported the prevalence of microbial respiratory infections as secondary infections in patients with COVID-19 [21].

Most microbial infections were more prevalent in males and the elderly over 45-years of age. This is partly explained by an attenuation of the inflammatory response by sex hormones in females [22]. Bereshchenko et al. [23] declared that infectious disease incidence is often male-biased due to differences in sex hormones and genetic architecture. The differences in the distribution of infections among different patients’ ages could be related to the strength of the immune system response, which would be expected to decrease in elderly patients [24]. On the other hand, the majority of studies concluded the predominance of female UTI, as compared to male UTI, as in this study, in which UTI is known as the disease of females. The main reason might be an anatomical predisposition compared to males, which allow bacteria access to the bladder as well as poor personal hygiene [25, 26].

Analysis of the data from the current study revealed that uropathogenic *E. coli* was the most frequently identified in urine specimens. In line with our study, Seifu and Gebissa [27] reported Gram-negative bacteria as the predominant species in patients with UTIs. Moreover, other studies found that *K. pneumoniae*, *P. mirabilis*, *S. saprophyticus, E. faecalis*, group B *Streptococcus* (GBS), *P. aeruginosa*, *S. aureus*, and *Candida* spp. are particularly relevant as hospital-acquired and catheter-associated infectious agents [28].

Our findings also showed that *Acinetobacter* spp., *P. aeruginosa*, *Klebsiella* spp., and *Serratia* spp. were the most predominant sputum and chest COVID-19 patient isolates. Previous studies by Sharifipour et al. [29] focused on secondary infection in COVID-19 respiratory patients and found that *A. baumannii* was the most common pathogen, followed by *S. aureus*. On the other hand, other studies in 2014 and 2018 on non-COVID-19 patients reported *A. baumannii* in respiratory patients and were associated with other bacteria, including *P. aeruginosa, Stenotrophomonas maltophilia, S. aureus, Enterococcus spp.*, and *K. pneumonia* [30]. In line with our results, *Candida* spp. was the most prevalent yeast isolate in respiratory samples and colonized the lower respiratory tract of mechanically ventilated patients [31].

In blood specimens, *S. aureus* and CON *Staphylococcus* spp. were the most commonly encountered Gram-positive bacteria, as in the study of Deku et al. [32], followed by Gram-negative bacteria and *Candida* spp. These findings were supported by Haddadin et al. [33] study, which found that *C. albicans* was the most common fungus involved in blood infections. In contrast to the present study, Khurana et al. [34] found that *Acinetobacter* spp. and *Klebsiella* spp. were the most common pathogens in bloodstream infections.

In wound infection, *S. aureus*, CON *S. epidermidis*, and *S. pyogenes* were the most frequently identified Gram-positive bacteria in the present study. Likewise, a prior study reported that *S. aureus* was the leading cause of wound infections, followed by *P. aeruginosa*, *Bacillus* spp., *E. coli*, *Candida* spp., and CON *Staphylococcus* spp. [35]. In comparison, other investigations found Gram-negative bacteria were the dominant in wound infection [36].

Considering the resistance pattern in Gram-negative bacterial isolates, *Acinetobacter* spp. from COVID-19 patients were extreme MDR isolates and showed complete resistance to most antibiotics, including *β*-lactams, cephalosporins, fluoroquinolones, and carbapenems, as reported in Sharifipour et al. [29] study, with the exception of glycylcycline (no resistance rate reported). However, lower rates of resistance to ceftazidime of 52.2% were recorded in a prior study [37]. In line with our study, a previous study by Namiganda et al. [38] reported that *A. baumannii* pulmonary strains were completely resistant to amikacin, ciprofloxacin, cotrimoxazole, ceftazidime, and piperacillin antibiotics, while 19% of isolates were sensitive to imipenem. Likewise, *S. rubidaea* and *S. liquefaciens* from COVID-19 patients were also extreme MDR isolates, and these were supported by the study of Namiganda et al. [38]. *Serratia* spp. isolates in our study showed high antimicrobial resistance levels when compared to Agyepong et al. [39] study. Early reports from Wuhan, China, indicated that half of the patients who died from COVID-19 developed secondary bacterial infections due to the high consumption of antibiotics during this viral pandemic [40, 41].

On the other hand, *H. alvei* and *K. ozaenae* were also extreme MDR strains and resisted all classes of antibiotics used, except for glycylcycline in *K. ozaenae*. However, Abbott et al. [42] reported different results and revealed *H. alvei* was susceptible at different rates to aminoglycosides, cephalosporins, monobactams, quinolones, and carbapenems. Moreover, another study reported *H. alvei* was resistant at different rates to amoxicillin (35%), cefoxitin (35%), ceftazidime (50%), and amikacin (40%), while it showed complete sensitivity to chloramphenicol [43]. In line with our study, Ghenea et al. [44] isolated *K. ozaenae* that was completely resistant to amoxicillin, ceftazidime, cefotoxime, amikacin, tetracycline, naldixic acid, erythromycin, and trimethoprim but sensitive only to imipenem and gentamicin, which contrasts our results.

*K. pneumoniae* clinical isolates in our study were extended spectrum *β*-lactam (ESBL) isolates, and these results were supported by the study conducted by Nirwati et al. [45] who found that *K. pneumoniae* was resistant to various antibiotics, including ampicillin, cefazolin, and cefuroxime, while amikacin, carbapenems, and piperacillin-tazobactam were the most favorable profile for treatment. The majority of the *E. coli* in our study was uropathogenic and showed resistance to penicillin, ampicillin, cefixime, and chloramphenicol antibiotics with different rates of 100, 97, 89, and 46%, respectively, which were consistent with previous studies [46, 47]. Additionally, *E. coli* showed good sensitivity to amikacin, glycylcycline, carbapenems (ertapenem and meropenem), ceftazidime-avibactam, ceftolozane/tazobactam, cefoperazone/sulbactam, and piperacillin/tazobactam, as reported by Scudeller et al. [48] study.

*E. gergoviae* isolates in our study were susceptible to most antibiotics, which contrasts with a previous study conducted by Friedrich et al. [49], who isolated *E. gergoviae* from bloodstream infections that were resistant to cefepime, carbapenems, piperacillin-tazobactam, aztreonam, and trimethoprim-sulfamethoxazole.

The high resistance rates of *P. mirabilis* isolates in the present study towards penicillin, ampicillin, sulphamethoxazole/trimethoprim, norfloxacin, ofloxacin, and gentamicin were reported to be at higher rates than in the Mirzaei et al. [50] study. On the other hand, *P. mirabilis* were completely sensitive to cephalosporins in our study; however, different resistance rates towards third-generation cephalosporins were reported in a previous study [51].

*P. aeruginosa* clinical isolates in the present study showed resistance rates of 100% towards penicillins, cefixime, chloramphenicol, and sulphamethoxazole/trimethoprim, and this was consistent with Motbainor et al. [52] findings. On the other hand, a low resistance rate to ceftazidime was reported in other studies in Ethiopia and Qatar, which were consistent with our results [53, 54]. The rates of *P. aeruginosa* resistance towards carbapenems (meropenem 50%) in the present study were coherent with other studies that documented resistance rates of 54, 45.5, and 41.7 in Bhatt et al. [55], Motbainor et al. [52], and Solomon et al. [53], respectively.

Considering the resistance pattern in Gram-positive bacterial isolates, about half of *S. aureus* were MRSA strains; these results were consistent with Taylor and Unakal [56] study. On the other hand, *S. aureus* reported a lower resistance rate of 13.4% towards oxacillin in Yılmaz and Aslantaş [57] reports that contrast our results. The ampicillin resistance rate (83.0%) in our study was consistent with the resistance rate findings of Yılmaz and Aslantaş [57] and Li et al. [58] studies but inconsistent with Gu et al. [59] study, which reported 49.2% resistance rates towards ampicillin and 17% towards gentamicin.

The resistance rate results of CON *Staphylococcus* in our study were supported by the study conducted by Xu et al. [60], who found that CON *Staphylococcus* isolates had high resistance rates to penicillin (94.7%), moderate resistance to oxacillin (52.6%), and low resistance to sulphamethoxazole-trimethoprim (33.9%). Furthermore, a low resistance rate of CON *Staphylococcus* towards linezolid has been reported in other studies that support our results [61]. On the other hand, high resistance rates to ciprofloxacin and amikacin were recorded in Adamus-Białek et al. [62] study, while low resistance rates towards clindamycin were found in Yılmaz and Aslantaş [57] study, which contrast our findings.

*Streptococcus* resistance rates of 33% towards penicillin and ampicillin in our study were relatively high compared to Rerambiah et al. [9] study. Penicillins are considered the first choice for the treatment of streptococcal infections [9]. More than 10% of patients reported an allergy to penicillin, leading to the use of macrolides as an alternative drug. Thus, the rates of macrolide resistance among *Streptococcus* spp. increased in North America [63].

Regarding the resistance pattern in *Candida* spp. clinical isolates, they showed high resistance rates to azole drugs. This resistance to azole drugs may be increased due to their general and long-term use in the treatment of *Candida* spp. [64]. The increase in *Candida* resistance to fluconazole is a matter of great concern, as it is the most commonly used azole for the treatment of candiduria [65]. On the other hand, all *Candida* isolates in our study were completely sensitive to polyene antifungal medication (nystatin), as reported in a previous study [66]. These results suggested nystatin could be used as an alternative drug for the treatment of azole-resistant *Candida* infections [67].

Finally, the high rates of resistance to azole drugs and most of the antibiotics used could be due to indiscriminate use, while the low rates of resistance to glycylcycline and nystatin antimicrobials could also be due to low prescription by physicians or low availability in different countries. The variation in results may be a result of the type and frequency of antibiotics used in different countries.

## Conclusions

There is an increase in the proportion of resistant Gram-negative, Gram-positive, and *Candida* spp. microbial isolates to most commonly prescribed antimicrobials. The high resistance rates of *Acinetobacter* spp., *Serratia* spp. (secondary microbial infection from COVID-19 patients), *H. alvei*, and *K. ozaenae* to all used antibiotic classes except glycylcycline are a major concern that portends an inevitable catastrophe. Guided prescriptions of antimicrobial agents should be implemented and controlled in hospitals to avoid the development of new generations of highly resistant microbial infections. Glycylcycline has been recommended for the treatment of MDR Gram-negative and Gram-positive bacteria, while nystatin has been recommended for the treatment of *candida* infections. Finally, recording the pathogens responsible for different infections and their antimicrobial resistance profiles, conducting an annual count of them, and continuous monitoring of antibiotic usage are vital to curbing existing microbial infections and identifying antimicrobial resistance patterns.

## Data Availability

All information created or analyzed during the present study are included in the manuscript.

## List of abbreviations

CON: Coagulase Negative
CONS: Coagulase Negative Staphylococci
ESBL: Extended spectrum *β*-lactam antibiotic
MDR: Multidrug resistant
MRSA: Methicillin-resistant *Staphylococcus aureus*
UTIs: Urinary tract infections
